# Uptake of exogenous estrogen as a differential diagnosis of ovarian-remnant-syndrome in a bitch: a case report

**DOI:** 10.1186/s12917-021-02923-9

**Published:** 2021-06-25

**Authors:** Sebastian Ganz, Axel Wehrend

**Affiliations:** Clinic of Obstetrics, Gynecology and Andrology of Large and Small Animals with Ambulatory Service, Faculty of Veterinary Medicine, Justus-Liebig-University, Giessen, Germany

**Keywords:** Ovarian remnant syndrome, Bitch, Estrogen, Uptake

## Abstract

**Background:**

Clinical signs of heat in bitches that have been previously spayed are often associated with the presence of ovarian remnant syndrome. The inclusion of exogenous estrogens as a differential diagnosis in this regard is often ignored and may lead to misinterpretation of the case.

**Case presentation:**

Herein, we report a case of exogenous estrogen exposure over several months to a 6.5-year-old spayed crossbred bitch, weighing 8.4 kg. The bitch presented in the clinic because of suspected ovarian remnant syndrome. Castration was performed within the first 6 months after birth. Important endocrine parameters measured at the first appointment were Anti-Müllerian hormone (< 0.01 ng/mL), progesterone (0.36 ng/mL), estradiol-17ß (20.7 pg/mL), and luteinizing hormone (< 0.1 ng/mL). After an extensive conversation with the owner, it was revealed that she was using an estrogen spray because of severe menopausal symptoms. After the owner stopped using this spray, the symptoms of the bitch disappeared.

**Conclusion:**

Therefore, the uptake of estrogens should be a differential diagnosis for symptoms of the ovarian remnant syndrome. A detailed anamnesis is crucial to identify the source of estrogen in the environment of the affected bitch.

## Background

In bitches with clinical signs of heat despite previous ovariectomy or ovariohysterectomy, incomplete castration is often suspected [1]. Incomplete castration is characterized by remnant ovarian tissues in a bitch after surgical castration [2]. Clinical presentation of bitches with the ovarian remnant syndrome vary greatly and can occur after a few weeks to several years [3]. Typically, recurring signs of heat, such as bloody discharge or swelling of the vulva, are observed [3]. However, symptoms not associated with heat, such as of pseudopregnancy may also occur [3]. Temporal variance in the occurrence of these clinical signs results from the fact that it takes different periods of time for the remnant ovarian tissue to revascularize, and the sexual cycle to be resumed [4]. There are various options available for diagnosis of the ovarian remnant syndrome. Exfoliative vaginal cytology is considered to play an important diagnostic role owing to its simple and inexpensive procedure [3]. More complex and objective diagnostic modalities are measurements of serum progesterone and estrogen concentrations in peripheral venous blood samples, concentration of luteinizing hormone, or Anti-Müllerian hormone (AMH) [3, 5]. If the bitch is diagnosed with ovarian remnant syndrome, surgery is indicated to remove the remnant tissue because of the possible development of endocrine-active cysts or a granulosa cell tumor from the original ovarian remnant or due to clinical signs related to ovarian tissue [6]. Besides the ovarian remnant syndrome, differential diagnoses for the occurrence of persistent or irregular heat signs should be considered. In intact bitches, the occurrence of endocrine-active ovarian cysts or hormonally active ovarian tumors should be mentioned [7, 8]. In pre-reported castrated bitches the intake of exogenous estrogens must also be considered [9]. The treatment of symptoms of menopause with estrogen preparations has become increasingly prevalent in human gynecology in the past decades [10], and these preparations are applied to the skin, primarily the forearm. The active ingredient is absorbed via the lipid metabolism of the skin into the body. This means that the dogs come into direct contact with the active ingredient by getting petted [10]. This can be a cause of signs of permanent or of irregular heat in the exposed bitches.

## Case presentation

### First examination (January 10, 2019)

**Fig. 1:**
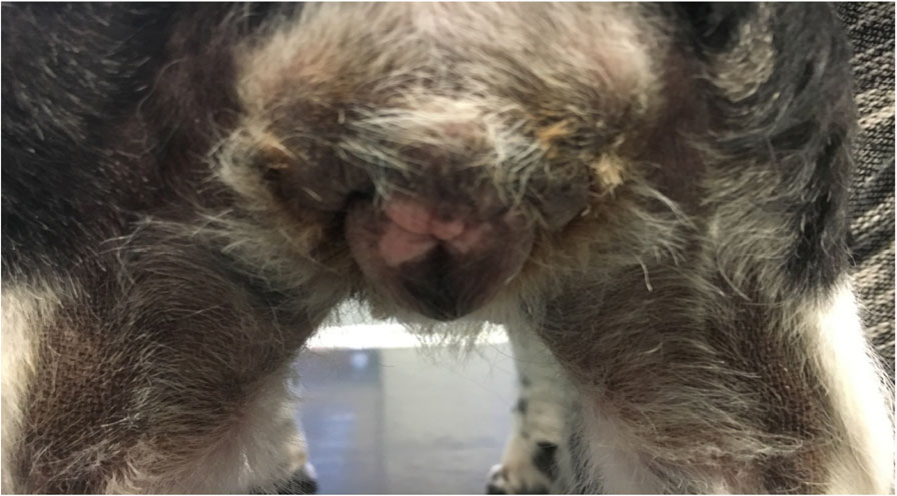
Edema of the vulvar and the perivulvar region

**Fig. 2:**
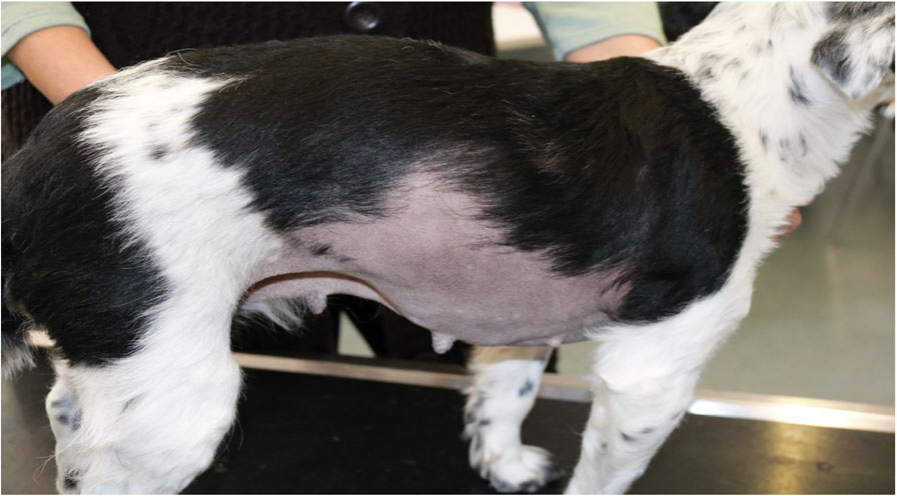
Severe alopecia the the side of the body

A 6.5-year-old spayed crossbred bitch, weighing 8.4 kg was presented because of recurrent signs of heat over the last 1.5 years. During this period, the bitch showed swelling of the vulva and vaginal discharge at irregular intervals with only few weeks without any signs of heat. After the first signs of alopecia at both flanks of the body occurred, the dog owner consulted a local veterinarian practitioner 6 weeks prior to the first appointment at our clinics. She was referred to the clinic of obstetrics of the Justus-Liebig-University in Giessen by the local vet for a detailed gynecological examination because of a suspected ovarian remnant syndrome. The bitch was presented with a good general condition. All parameters of the clinical examination were within the physiological ranges. The bitch showed obvious pruritus of the vulva and inguinal area with a prominent edema of the vulva (Fig. 1). Additionally, there was severe alopecia on both sides of the body and clear, serous vaginal discharge (Fig. 2). The mammary glands of the dog were without noticeable abnormal findings. In the gynecological examination, a vaginal cytology was performed. It showed predominantly nuclear and anuclear superficial cells. These cell types made up 90% of all cells of the smear. Only a few intermediate cells with less than 10% of the overall cell population could be detected. These findings indicated a strong estrogen influence. A vaginoscopy could not be performed because of the strong defense reaction of the dog. In addition an abdominal ultrasound was performed. In the caudal abdominal cavity, a prominent cervical stump was obvious. Caudal to either kidney, there were no signs of ovarian tissue.

To rule-out some remaining ovarian tissue, a blood sample was obtained to determine the following parameters: concentration of AMH, luteinizing hormone (LH), progesterone, estradiol-17ß, activity of alanine aminotransferase (ALT), and concentrations of creatinine and urea. The AMH, progesterone and LH concentration were low and concentration of estradiol-17ß was slightly elevated. As part of the routine in the clinic, ALT, creatinine and urea was analyzed. The concentration of the kidney-related parameters was slightly above the physiological range, whereas the concentration of ALT was within the physiological range. Additionally, a blood count was performed to rule out a possible suppressive effect on the bone marrow in the course of a long-term exposure of estrogens. All parameters of the blood count were within the physiological ranges. Table 1 summarizes the results. A detailed counselling interview revealed that the owner was using an estradiol spray for nearly 1.5 years because of menopause. This spray is typically put on both the forearms. The owner was recommended to stop using the spray or to use it on body parts that the dog is not exposed to, e.g. the inner thighs or back.

**Table 1:** Blood parameters of the first examination

Parameter	Concentration	Reference range
AMH	< 0.01 ng/mL	< 0,06 ng/mL for spayed bitches^a^
Progesterone	0.36 ng/mL	< 1,5 ng/mL basal level
Estradiol-17ß	20.7 pg/mL	< 15 pg/mL basal level
LH	< 0.1 ng/mL	< 1 ng/mL for intact bitches apart from the LH-peak
Creatinine	169 μmol/L	0–159 μmol/L
ALT	< 55 IU/L	0–39 IU/L
Urea	9.72 mmol/L	0–9 mmol/L

*AMH* Anti-Müllerian hormone, *LH* Luteinizing hormone, *ALT* Alanine aminotransferase; ^a^a human-based kit validated for dogs was used

### Second examination (February 6, 2019)

The owner of the dog told that after the first appointment she immediately stopped the use of the estrogen spray. Two weeks after cessation of her estrogen treatment, there was no vaginal discharge of the bitch and the edema of the vulva seemed to be less pronounced. At the second examination the findings of the owner could be retrace. The edema of the vulva showed clear regression but was still present. The examination revealed no vaginal discharge either. Vaginal cytology showed only intermediate cells. Nuclear and anuclear superficial cells had completely disappeared. The only symptom detected in the first examination that showed no improvement at the second appointment was the severe alopecia.

### Third examination (March 18, 2019)

**Fig. 3:**
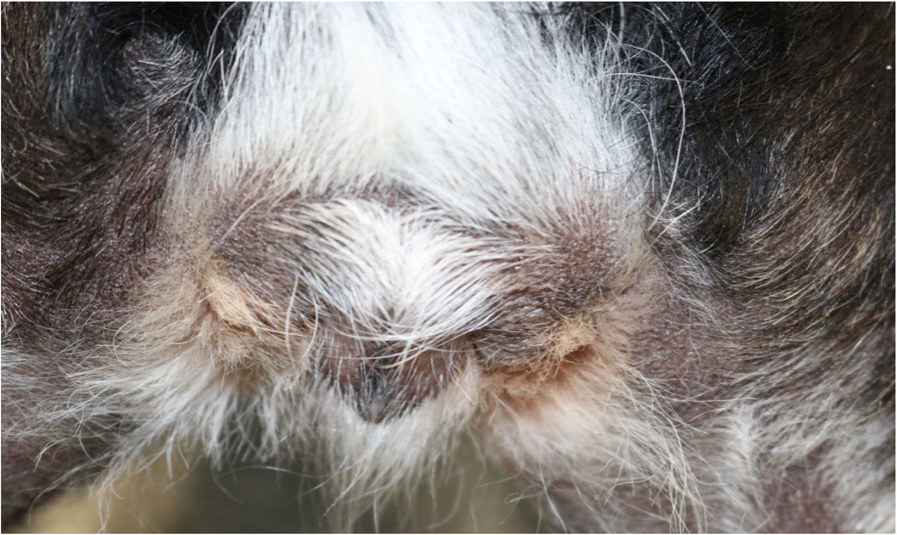
The edema of the vulva has completely disappeared

**Fig. 4:**
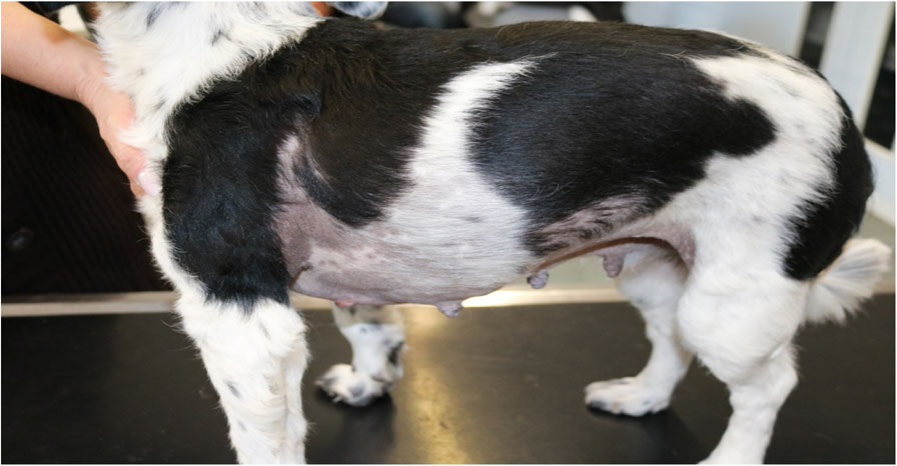
Regrowth of the fur was noticable

Three weeks after the second appointment the dog owner had noticed a complete regression of the vulvar edema. This observation was confirmed in the third examination. At that time the edema of the vulva had completely disappeared (Fig. 3). There was no vaginal discharge, and the cytology showed only parabasal and basal cells. The alopecia was still obvious, but regrowth of the fur around the teats on both sides of the body was noticeable (Fig. 4).

## Discussion and conclusion

Permanent or irregular signs of estrus in a bitch after castration could have different reasons [9]. The differential diagnosis includes incomplete castration (ovarian remnant syndrome) or the uptake of exogenous estrogens [11–14]. Usually after gonadectomy, bitches have higher concentration of gonadotropins (luteinizing hormone, follicle-stimulating hormone) in the circulation compared to intact bitches [15]. These findings result from the loss of negative feedback from the gonads to the pituitary gland and hypothalamus [15, 16]. Despite the loss of negative feedback of the pituitary–gonadal axis in some cases, there are overlapping results of serum gonadotropins between intact and castrated bitches because of on the one hand a lower concentration of gonadotropins together with an irregular secretory pattern in long term castrated bitches and on the other hand a physiological pulsatile secretion pattern of luteinizing hormone and follicle-stimulating hormone in the bitch in general [17]. Therefore, AMH can be a useful parameter to distinguish between spayed bitches and bitches with active ovarian tissue [5]. High concentrations of AMH in the peripheral blood circulation is an ideal marker for suspected ovarian remnant syndrome because AMH is secreted by the granulosa cells of the ovarian tissue exclusively [18], which means a high concentration of AMH correlates with the presence of ovarian tissues in bitches [4]. It should be mentioned that there is a rare possibility that the interpretation of the AMH concentration is false positive because of an antibody interference with the test when using a human based kit [19]. Depending on the test kit other studies found also low AMH concentration in intact bitches [5]. When comparing and interpreting the results of different studies it is important to know if a humane- or a canine-based kit was used. The canine-based kit has a superior ability to label the canine AMH molecule and therefore the values obtained with these kits tend to be higher compared to the human-based kit [20]. In this study a human-based kit validated for dog serum was used (LABOKLIN GmbH & Co. KG, Bad Kissingen, Germany). The threshold for a spayed bitch without ovarian tissue left behind when using the human-based kit as in our study is below 0.06 ng/mL and above 0.09 ng/mL for intact bitches [21]. The diagnosis of permanent estrus signs due to an uptake of exogenous estrogens requires a detailed anamnesis and conversation of the veterinarian with the owner. In the present case, the low concentration of AMH indicated that no ovarian tissues had remained in the bitch. This finding was confirmed by the low concentration of progesterone in the blood sample. The determination of the concentration of progesterone to investigate whether ovarian tissues were present or not is only reliable when the bitch is not in pro- or anestrus because in these periods of the cycle the progesterone concentrations are basal with values below 1.5 ng/mL whereas the concentration of AMH does not depend on the stage of the reproductive cycle [5]. In contrast to these interpretations, the concentration of the luteinizing hormone was low, which in most cases indicates that the bitch is not castrated because in intact bitches, there is negative feedback from the gonads to the pituitary gland and hypothalamus. This finding can be explained with the pulsatile secretion pattern of luteinizing hormone mentioned above or with a negative feedback of the exogenous estrogens on the secretion of LH of the pituitary gland. The slightly elevated concentration of estradiol-17ß shows an influence of estrogen without being specific. The elevated concentration of kidney-related parameters could be a result the influence of estrogens but is also not directly related to it. Because the red blood count showed no pathological abnormalities a chronic estrogen toxicity and a suppressive effect of the bone marrow could be ruled out. The interpretation of the measured laboratory parameters combined with the clinical examination and discussion with the owner led to the conclusion that the reason of the high concentration of estradiol-17ß in the peripheral blood sample of the bitch was an exogenous source of estrogens.

Potential sources are preparations with estrogens that are applied to the forearm of women with severe menopausal symptoms. As a result, direct exposure of the dog occurs. Typically, small or toy breeds are affected by clinical signs of permanent or irregular heat due to exogenous estrogen since these breeds are increasingly carried on the arms, thus coming into direct contact with the active substance, such as by licking. Dogs that are permanently exposed to estrogens may show symptoms over a prolonged period of time that are usually associated with proestrus or estrus [9]. In addition, estrogen can lead to intoxication because it is well known that permanent exposure to estrogens can have a suppressive effect on the bone marrow and so on cytogenesis, which may result in first regenerative anemia which can develop into a non-regenerative anemia or even pancytopenia and death [9]. In addition, this prolonged exposure can result in the development of a stump pyometra due to proliferation of the uterine mucosa of the cervical stump [9]. After exclusion of pathological processes and the ovarian remnant syndrome, the possibility of uptake of exogenous estrogens must be considered to provide the patient with permanent relief. Therefore, a detailed anamnesis is an indispensable instrument to be able to react adequately in such cases and to avoid unnecessary surgical intervention. The owner must be informed about the effects of the use of such preparations on the dog. If possible, the treatment should be discontinued completely. If not, the affected patients should visit the treating human gynecologist and ask about the possibility of alternatives, e.g. preparations that can be taken orally. Another possibility is to apply the spray to areas of the body with which the dog does not come into direct contact. After discontinuing the medication or switching to medication that is not applied to the skin, the symptoms of the bitches should decrease within a few weeks and eventually disappear completely. It is recommended that affected bitches be reappointed to the practice or clinic to check the outcome through a repeated gynecological examination.

This report shows that exogenous uptake of postmenopausal human estrogen drugs through the owner can be a differential diagnosis to signs of the ovarian remnant syndrome and should be ruled-out when a dog presents with clinical signsmentioned above.

## Data Availability

All data generated or analyzed during this study are included in this published article.

## Abbreviation

AMH: Anti-müllerian-hormon
